# Double Emergency: A Case of Concurrent Heparin-Induced Thrombocytopenia and Acute Promyelocytic Leukemia

**DOI:** 10.7759/cureus.58927

**Published:** 2024-04-24

**Authors:** Anastasia Schuldt, Mohamed Samour

**Affiliations:** 1 Hematology and Medical Oncology, Geisinger Medical Center, Danville, USA; 2 Hematology and Medical Oncology, Geisinger Wyoming Valley, Wilkes-Barre, USA

**Keywords:** thrombocytopenia, leukemia, promyelocyte, heparin, acute promyelocytic leukemia, heparin-induced thrombocytopenia

## Abstract

A 48-year-old woman presented to the hospital with acute pulmonary embolism in the setting of presumed apixaban failure and was transitioned to heparin. Rapidly progressive pancytopenia prompted workup with suspicion for heparin-induced thrombocytopenia (HIT) as well as peripheral blood smear concerning for acute promyelocytic leukemia (APL). She was emergently started on non-heparin anticoagulation and transferred to start APL-directed treatment. Both HIT and APL were confirmed with serotonin release assay (SRA) and promyelocytic leukemia/retinoic acid receptor alpha (PML-RARA) fusion assay, respectively. We present this case to remark on the novelty of these two acute diseases occurring together. Each of these entities is a hematologic emergency requiring immediate treatment before disease confirmation. We explore the mechanisms by which HIT occurs and outline the parameters for managing APL in the acute setting. Furthermore, this case serves to examine the treatment considerations for needing to carefully balance the thrombotic and hemorrhagic risk of both HIT and APL, which are clinically well-known for coagulopathy. Fortunately, HIT in this patient recovered on anticoagulation without bleeding or worsening thrombosis. Furthermore, following induction and consolidation treatment for APL, she remained negative for residual disease.

## Introduction

Both acute promyelocytic leukemia (APL) and heparin-induced thrombocytopenia (HIT) are independent hematologic emergencies but are often not managed simultaneously. Each of these entities has a hematologic risk of hemorrhage and thrombosis with careful medical management needed to mediate these complications. Prior cases of HIT occurring in the hematologic malignancy population exist mostly in the form of retrospective analyses and case reports [1-3]. Especially in APL, there remains a paucity of evidence regarding the approach and management of HIT, and at the time of this case report, we have encountered no prior published case of APL and HIT presenting concurrently. In this review, we present a case of a woman presenting acutely with HIT and APL. This case is not only novel in its presentation but also affords the opportunity to examine her management of bleeding and thrombosis risk during APL induction and consolidation treatment and to explore the outcomes and complications of managing these conditions concurrently.

In brief, HIT occurs as an immune-mediated reaction of antibodies against the heparin-platelet factor 4 (PF4) complex. This results in thrombocytopenia but also presents a high risk of both venous and arterial thrombosis and requires ongoing non-heparin anticoagulation to mediate this risk. The probability of HIT may be predicted by the presence of heparin-PF4 antibodies in the correct clinical pattern but is confirmed with functional assays such as a serotonin release assay (SRA) [4,5].

APL, on the other hand, is a hematologic malignancy presenting often with increased peripheral blood blasts, pancytopenia, and coagulopathy. During acute management, special attention is needed with close clinical and laboratory monitoring to avoid the risk of fatal hemorrhagic complications. Like HIT, the treatment of APL is emergent and starts when the disease is suspected while confirmatory testing is pending. APL is confirmed by the presence of the promyelocytic leukemia/retinoic acid receptor alpha (PML-RARA) fusion protein [6-8].

## Case presentation

A 48-year-old woman with essential hypertension, generalized anxiety disorder, and morbid obesity presented to the emergency department with chest heaviness and cough for one month. Around the time of symptom onset, she tested positive for coronavirus disease 2019 (COVID-19), and managed conservatively at home. She did not have any laboratory workup at that time, but a routine complete blood count (CBC) performed three months prior revealed no abnormalities. Two weeks later she received a 10-day course of cefdinir for an ear infection and steroids due to persistent cough, but her cough progressed, and she developed scant hemoptysis. She suspected she may have aspirated some cherry pie.

In the emergency room, she was afebrile but tachycardic with a heart rate of 111 beats per minute, blood pressure of 117/75 mmHg, with SpO2 of 97% on room air. Electrocardiogram revealed normal sinus rhythm without ST changes and troponins were normal. Computed tomography (CT) chest revealed a large left lower lobe pulmonary embolism with associated pulmonary infarct. She was discharged from the emergency department on apixaban 10 mg twice daily with anticipated hematology follow-up. Her CBC at that time revealed developing leukopenia and anemia with WBC 2.04 K/uL, hemoglobin (Hb) 11.0 g/dL, and platelets 159 K/uL. The embolism was deemed to be driven by a recent COVID-19 infection. Her other risk factors for venous thromboembolism (VTE) included obesity and the use of intramuscular depot medroxyprogesterone [9]. She had no prior history of thrombosis. 

The following week, she presented again with worsening left-sided pleuritic pain. Repeat CT chest now demonstrated bilateral pulmonary emboli. Bilateral lower extremity duplex ultrasound was negative for deep vein thrombosis. Suspecting apixaban failure related to obesity, she was started on continuous heparin infusion. Notably on presentation and prior to starting heparin, she was now pancytopenic with WBC 1.57 k/uL, absolute neutrophil count 0.55 k/uL, hemoglobin 9.7 g/dL, and platelets 95 k/uL. A peripheral smear review performed three days after admission demonstrated pancytopenia with normocytic anemia. There was no commentary on leukocyte morphology. Imaging revealed increasing left pleural effusion and infiltrate, and in the setting of worsening respiratory failure, this prompted diagnostic and therapeutic thoracentesis. A total of 1.4 L of dark red pleural fluid was collected with negative cytology for malignant cells. She remained on empiric antibiotics for suspected pneumonia as well as urinary tract infection and received filgrastim for two days due to the concern of developing severe sepsis in the setting of neutropenia. A summary of her laboratory results is displayed in Table 1.

**Table 1:** Patient's laboratory values from peripheral blood on presentation OD = optical density; PF4 = platelet factor 4

Lab Name	Patient's Lab Value	Normal Reference Range
White blood cells	1.57 k/uL	4.00-10.80 k/uL
Hemoglobin	9.7 g/dL	12.0-15.3 g/dL
Hematocrit	27.2%	36.0-45.2%
Platelets	92 k/uL	140-400 k/uL
Absolute neutrophil count	0.55 k/uL	1.80-7.70 k/uL
Heparin PF4 antibody	2.497 OD	< 0.400 OD
Serotonin release assay, unfractionated heparin	93%	< 20%
Vitamin B12	369 pg/mL	232-1245 pg/mL
Folate	6 ng/mL	> 4.5 ng/mL
Lactate dehydrogenase	678 U/L	< 250 U/L
Haptoglobin	375 mg/dL	10-200 mg/dL

**Figure 1:**
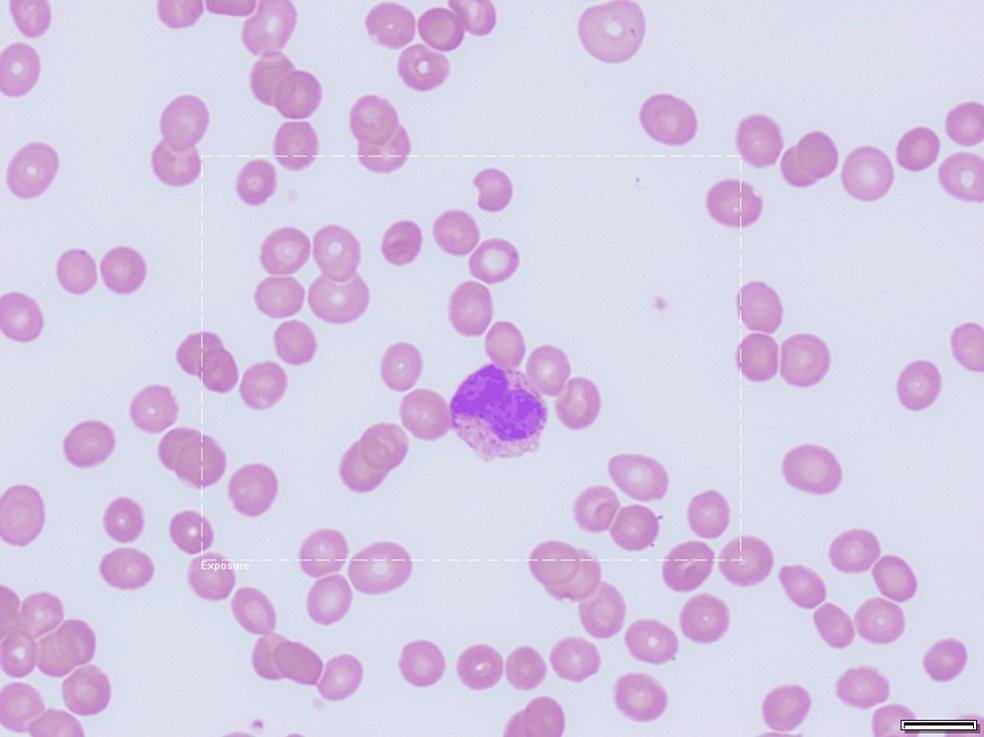
Promyelocyte on peripheral blood smear, 100X The typical appearance of a promyelocyte is a large cell with bluish, granule-filled cytoplasm. The granules contain myeloperoxidase. The nucleus of a promyelocyte is sometimes indented or bilobed [8]. In this image, the dotted white rectangle represents the exposure field used to auto-adjust the camera light to the field of interest on our slide.

Thrombocytopenia progressed. Vitamin B12, folate, and hepatitis/HIV screen were normal and hemolysis workup was negative. 4T score for the pre-test probability of HIT was four, indicating intermediate risk level. The Heparin PF4 antibody test resulted positive at 2.479 OD, so she received fondaparinux while awaiting confirmatory functional testing for HIT. Additionally, a repeat peripheral smear review revealed an increased number of promyelocytic appearing cells concerning for APL (Figure 1). She was promptly transferred for inpatient hematology care to start all-trans retinoic acid (ATRA), which was unavailable at her presenting hospital due to supply shortages.

Following the transfer, anticoagulation continued with argatroban for suspected HIT, with the diagnosis later confirmed by positive SRA. She continued on argatroban drip while also managing possible APL with ATRA, close monitoring, and steroids for differentiation syndrome prophylaxis. Flow cytometry demonstrated an immature myeloid population (63% on peripheral blood, 72% on bone marrow aspirate), positive for slight dim CD45 and negative for CD34. When fluorescence in-situ hybridization (FISH) for PML-RARA t(15;17) on bone marrow specimen confirmed the diagnosis of APL, she started arsenic trioxide (ATO) in addition to ATRA. The PML-RARA fusion transcript was also detected on peripheral blood by reverse transcriptase polymerase chain reaction (RT-PCR), which was sent from her presenting hospital before transfer. Karyotype was also consistent with 46XX with t(15;17) identified in 19 of 21 assessed cells. Acute myeloid leukemia gene panels were unremarkable for additional mutations.

Thrombocytopenia recovered on treatment, and she transitioned back to apixaban briefly, later to warfarin with fondaparinux bridge due to concerns of obesity and prior apixaban failure. Following ATRA/ATO chemotherapy induction, bone marrow showed remission. She was discharged from the hospital and continued on ATRA/ATO consolidation.

At the time of diagnosis, quantitative PCR for PML-RARA showed 2991.379 normalized copy number (NCN). One month after induction, this reduced to 0.570 NCN on peripheral blood and thereafter remained undetectable on repeat measurements taken about six weeks apart.

**Figure 2:**
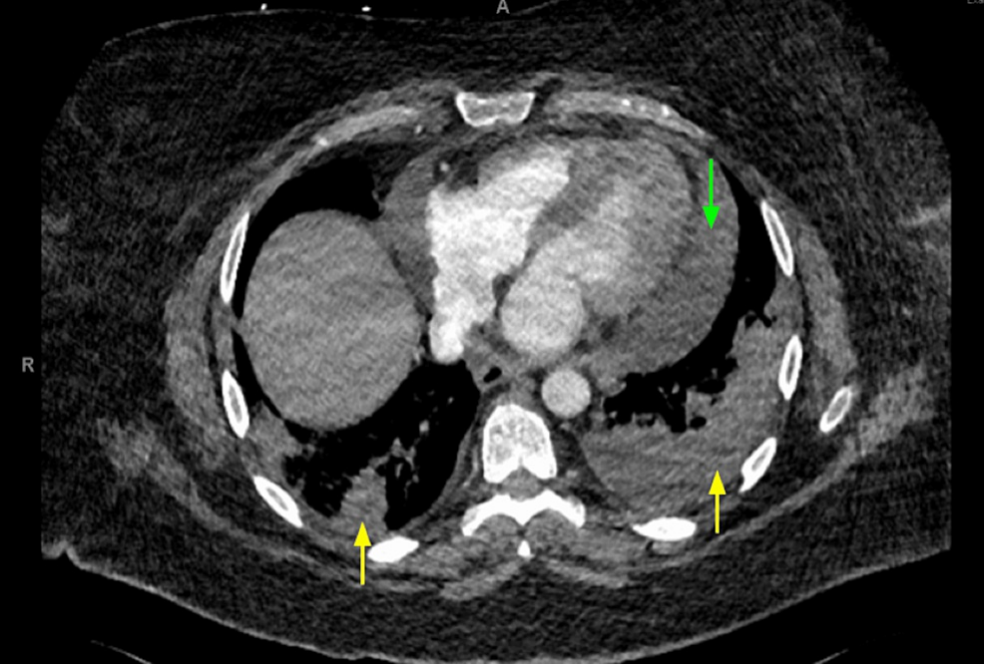
Pericardial effusion and pulmonary infarcts on CT chest Our patient with acute promyelocytic leukemia and prior pulmonary embolism presented with increasing dyspnea and fatigue. CT of the chest demonstrated pericardial effusion (green downward arrow) as well as evolving bilateral pulmonary infarcts (yellow upward arrows). She was managed with pericardiocentesis, ongoing therapeutic anticoagulation, and supportive treatment including supplemental oxygen.

Unfortunately, she was readmitted twice with hypoxemia. Her first readmission involved pericardial effusion without tamponade (Figure 2), managed with pericardiocentesis of a small amount of serosanguinous fluid followed by a prolonged steroid taper. Her second readmission involved increasing right-sided pleural effusion, managed with chest tube placement. This effusion was exudative but negative on culture and cytology. She required ongoing three liters of supplemental oxygen. Outpatient right heart catheterization was negative for pulmonary hypertension, and she was later weaned off oxygen. It is possible that these complications including pericardial and pleural effusion may have been signs of differentiation syndrome, and this may be further supported by her symptomatic improvement on steroids. However, differentiation syndrome was not addressed in her chart during this hospital stay. The cardiology team recommended prolonged prednisone taper as they suspected the effusion was inflammatory in nature and related to leukemia. She continued on ATRA/ATO consolidation therapy during admission.

She completed four cycles of consolidation ATRA/ATO and currently maintains molecular remission on PML-RARA quantitative PCR. Furthermore, ten months following diagnosis of HIT her heparin PF4 antibodies are negative at 0.077 OD. She still suffers from deconditioning but is off supplemental oxygen now for three months and is looking to return to work. She remains on warfarin with an international normalized ratio (INR) goal of 2-3, anticipated to complete at least one year of anticoagulation and then assess thrombosis risk for possible discontinuation. If anticoagulation is discontinued, we will consider a hypercoagulable workup off anticoagulation. At the time of writing this article, she is almost one year from her initial diagnosis of APL and remains on clinical observation.

## Discussion

Type II HIT is characterized by thrombocytopenia alongside a severe hypercoagulable state and is mediated by antibodies targeting the heparin-PF4 complex. This is unlike type I HIT (also known as heparin-associated thrombocytopenia (HAT)) which is mild, self-limited, and not immune-mediated [4]. In both literature and clinical practice, the general term HIT refers mostly to type II HIT.

Heparin-PF4 antibodies typically form within 5-14 days of heparin exposure, with the clinical and laboratory manifestation of HIT arising after this time period. However, a patient previously exposed to heparin within the last 100 days may still have heparin-PF4 antibodies present; in that setting, heparin may trigger thrombocytopenia immediately upon re-exposure [4].

How does HIT paradoxically cause hypercoagulability in the setting of thrombocytopenia? The answer relies on understanding the function of heparin and platelets. Heparin’s anticoagulant activity is not direct but occurs when heparin binds to antithrombin, which enhances antithrombin’s ability to inactivate thrombin (factor II) and factor X [5]. Mature platelet factor 4 (PF4) is a chemokine protein synthesized by megakaryocytes and stored in the alpha granules of platelets. PF4 has known physiologic functions in promoting blood coagulation and inflammation [4,5]. Activated platelets release PF4 which can bind to heparan, a heparin-like substance on endothelial cells [4]. When PF4 binds to heparin, heparin cannot bind to antithrombin and no longer acts as an effective anticoagulant. In HIT, IgG antibodies bind to the heparin-PF4 complex, and then the Fc receptor on these antibodies binds to platelets and triggers further PF4 release, thereby creating a positive-feedback cascade of platelet activation and resulting in platelet aggregation and thrombin generation [3,4,5]. Platelet transfusion is contraindicated during acute HIT [4]. PF4/heparin/antibody complex also activates monocytes, which causes increased expression of tissue factor, further promoting coagulation by activating factors IX and X [5].

Evaluating thrombocytopenia in the cancer population can lead to a broad differential diagnosis. Chemotherapy remains a common cause of thrombocytopenia. Furthermore, many cancer patients are exposed to heparin in the form of flushes. Severe HIT can produce schistocytes, which may place other microangiopathic hemolytic anemias in the differential [3].

Are cancer patients at an increased risk of HIT compared to non-cancer populations? Current studies suggest not. One retrospective analysis from MD Anderson Cancer Center estimated the risk of HIT among heparin-exposed cancer patients to be 0.24%, which is lower than the reported risk of HIT in the general population [2,4]. A reduced incidence of HIT may have been related to the inclusion of patients with lower-risk heparin exposures such as heparin flushes. When compared to solid malignancies, hematologic malignancies were less likely to have anti-heparin-PF4 complex antibodies. However, the dataset was limited and could not compare risk among specific malignancy types [2]. In a 2021 retrospective analysis of malignancy-associated HIT cases, the most common solid tumor malignancy was gastrointestinal cancer, and the most common hematologic malignancy was myeloproliferative neoplasm [1]. Among patients with HIT, malignancy does not appear to increase all-cause mortality. While patients with malignancy may have other confounding factors in the workup of thrombocytopenia, there is currently no recommendation regarding changes to HIT screening or confirmatory testing strategy in the cancer population [3].

In similarity to HIT, APL has its own considerations of management requiring immediate intervention with suspected diagnosis as well as careful lab monitoring and risk assessment. Like HIT, APL is a hematologic emergency. Upon APL suspicion, ATRA should be started while awaiting confirmation of diagnosis with PML-RARA fusion, after which the patient is started on ATO [6,8]. This sequence was abided in our case of standard risk APL. As ATRA and ATO are teratogenic, patients with childbearing potential should be advised against pregnancy. In patients presenting with high-risk APL (WBC > 10,000/uL), consideration can be made for cytoreduction with anthracyclines (idarubicin, daunorubicin) sometimes combined with cytarabine, or with gemtuzumab ozogamicin [6].

Techniques employed for identifying PML-RARA include FISH, PCR (reverse transcriptase PCR (RT-PCR) or real-time quantitative PCR (RQ-PCR)), or karyotype. PCR is notably important for the purposes of identifying the specific PML-RARA isoform and monitoring this isoform for future minimum residual disease (MRD) analysis. Over time, variants of RARA fusion outside of the classic PML-RARA have been identified, but there is still much to learn [6,8] Of note, some of these fusions, such as ZBTB16-RARA and STAT5b-RARA are less responsive to ATRA and may require treatment utilizing acute myeloid leukemia regimens. The clinical significance of identifying other mutations including NRAS, KRAS, WT1, and FLT3 as well as the role of next-generation sequencing testing is less clear, and at present these mutations do not have a marked impact on treatment or prognosis [6].

APL patients must be carefully followed for complications such as coagulopathy, differentiation syndrome, and other treatment-related toxicities. Coagulopathy in APL requires frequent lab monitoring and replacement of blood products to maintain fibrinogen above 100-150 mg/dL, INR below 1.5, and platelets above 30-50 k/uL. Due to risk of hemorrhage, care teams should avoid procedures if possible including central venous catheterization, lumbar puncture, or bronchoscopy. Leukapheresis is avoided due to central access risk [6]. Differentiation syndrome is a potentially fatal complication that occurs in about 25% of APL patients. Symptoms include weight gain, peripheral edema, shortness of breath, and acute kidney injury. When differentiation syndrome is suspected, patients should be started on steroids (dexamethasone 10 mg IV twice daily) until symptom resolution, ideally while continuing ATRA and ATO. ATRA and ATO may need to be held in the setting of severe organ dysfunction related to differentiation syndrome, such as renal failure requiring dialysis or respiratory failure requiring intubation [8]. While on ATO, the QT interval must be carefully monitored for prolongation [6]. Despite many clinical risks, APL is curable and highly responsive to therapy [8]. In our APL patient case, the concurrent presentation of HIT added yet another layer of complexity to a difficult hospital scenario.

Notably, our patient presented with pulmonary embolism prior to exposure to heparin, so her initial thrombotic episode was not related to HIT but may have been related to other thrombotic risk factors including recent COVID-19 and depot birth control. She started heparin upon apixaban failure with pulmonary embolism. As her neutropenia worsened, she received filgrastim for two days prior to hospital transfer to our hematology service. Initially, we suspected that the blast forms noted on smear review may have been related to filgrastim, but the presence of PML-RARA fusion confirmed APL. It may be possible that filgrastim could have accelerated the clinical presentation of the blast forms, but undoubtedly this was a true malignancy. In literature, filgrastim is safely used following induction in acute myeloid leukemia to help minimize post-treatment cytopenias and reduce the need for antifungal prophylaxis [7,10]. However, in our patient filgrastim was used before any cancer-directed treatment was started, and this case serves as a reminder to use caution with regard to the use of stimulating factors in pancytopenia yet undergoing workup.

Both HIT and APL require emergent treatment intervention while awaiting confirmatory diagnosis, and this was performed in our patient in the form of continuing anticoagulation for HIT as well as starting ATRA with close lab monitoring for APL. Due to worsening respiratory failure with pleural effusion, our patient underwent thoracentesis prior to diagnosis of either HIT or APL. One of the most difficult clinical determinations in this case was the role of transfusion. She received a platelet transfusion on three occasions prior to the transfer. Platelet transfusion is generally contraindicated in HIT as it may worsen the thrombotic mechanisms described earlier [4]. On the other hand, transfusion to maintain a platelet goal of 30-50 k/uL is often employed in APL, where coagulopathy and hemorrhage are major risks [6]. There is no perfect clinical solution here. Luckily, her platelets continued to rise on argatroban drip and ATRA, and she did not have any bleeding concerns in the acute setting. A case such as this can be a tenuous balance of disease - requiring anticoagulation without transfusion for HIT but also maintaining platelet goals to mitigate the bleeding risk of APL. Thankfully, the solution in this case was to maintain anticoagulation, carefully assess for bleeding, and proceed with APL-directed treatment with ATRA and ATO.

At present, the appearance of simultaneous HIT and APL appears novel. Current literature does not suggest a known correlation between these two entities, and it is possible that the relationship in this case may be rare but coincidental and less likely reflective of an underlying causative mechanism linking both disorders. Prior observational studies as noted above have reported a lower risk of HIT in a hematologic malignancy population [3].

## Conclusions

What occurred as the simultaneous presentation of two hematologic emergencies, HIT and APL, thankfully resulted in the resolution and remission of both entities with our patient clinically well and disease-free now almost a year out from diagnosis. We have yet to encounter another example of HIT and APL presenting concurrently in the literature, and this case offers the unique scenario of balancing two highly coagulopathic diseases. There does not appear to be an established correlation between HIT and APL so this may have been coincidental. It is possible that the key to success in this case was the emergent initiation of treatment for each disease upon presumed diagnosis followed by confirmatory clinical workup and advancing treatment once HIT and APL were confirmed. Treatment consisted of continuous argatroban infusion with transition to warfarin, careful monitoring of laboratory values, assessing for any clinical bleeding or worsening thrombosis, and continuing ATRA/ATO for APL. While APL is highly curable, the possibility of life-threatening acute complications may be enhanced by this presentation of simultaneous HIT. This unique case serves to help us examine the underlying coagulopathy mechanisms and the difficulty of balancing these risks. Most importantly, this case reinforces the importance of immediate intervention on suspected hematologic emergencies, as even high-risk scenarios may have promising outcomes with appropriate management.
